# Oxidative Stress in Association with Metabolic Health and Obesity in Young Adults

**DOI:** 10.1155/2021/9987352

**Published:** 2021-06-26

**Authors:** Grzegorz K. Jakubiak, Kamila Osadnik, Mateusz Lejawa, Sławomir Kasperczyk, Tadeusz Osadnik, Natalia Pawlas

**Affiliations:** ^1^Department of Pharmacology, Faculty of Medical Sciences in Zabrze, Medical University of Silesia, Jordana 38, 41-808 Zabrze, Poland; ^2^Department and Clinic of Internal Medicine, Angiology and Physical Medicine, Faculty of Medical Sciences in Zabrze, Medical University of Silesia, Batorego 15, 41-902 Bytom, Poland; ^3^Department of Biochemistry, Faculty of Medical Sciences in Zabrze, Medical University of Silesia, Jordana 19, 41-808 Zabrze, Poland; ^4^2nd Department of Cardiology and Angiology, Silesian Center for Heart Diseases, Marii Skłodowskiej-Curie 9, 41-800 Zabrze, Poland

## Abstract

**Introduction:**

Obesity is one of the most important public health problems in the world. Among obese people, there are those who, apart from excessive body weight, do not exhibit other metabolic dysfunctions, have a lower risk of developing cardiovascular diseases (CVDs), and show lower mortality. According to the theory, they are referred as metabolically healthy obese (MHO), in contrast to metabolically unhealthy obese (MUO). Metabolic disturbances occurring with obesity have been well established to be associated with oxidative stress.

**Aim:**

The purpose of this study was to analyse the association between selected anthropometric and biochemical parameters with oxidative stress in MHO, MUO, and normal weight young adults. *Material and Methods*. Individuals with age between 18 and 36 years with no history of chronic diseases and use of medicaments, with or without obesity, participated in the study. Complete blood counts, biochemical measurements, and parameters of oxidative stress such as total antioxidant capacity (TAC), total oxidative status (TOS), oxidative stress index (OSI), serum concentration of malondialdehyde (MDA), ceruloplasmin, thiol groups and lipid hydroperoxides (LPH), concentration of lipofuscin (LPS) in erythrocytes, and the activity of superoxide dismutase (SOD) were measured.

**Results:**

422 patients who met the inclusion criteria were enrolled in the study. Among the study participants, 208 people (49.29%) were offspring of patients with angiographically confirmed coronary artery disease. Among the participants, 16 patients have been included in the group of metabolically healthy obese (MHO) people and 61 patients in the group of metabolically unhealthy obese (MUO) people and 345 patients had normal body weight. Significant differences between metabolically unhealthy obese and normal weight patients, as well as between women and men, have been found.

**Conclusions:**

We showed significant differences in the selected parameters of oxidative stress between metabolically unhealthy obese (MUO) individuals and young volunteers with normal body weight as well as without significant medical history.

## 1. Introduction

Obesity is a significant risk factor for metabolic disorders leading to multiple diseases such as diabetes mellitus, cancer, and cardiovascular diseases [1]. It has been observed that there is a subgroup of patients, called metabolically healthy obese (MHO), with no metabolic abnormalities despite excessive body weight. Patients with metabolic disorders in the course of obesity belong to metabolically unhealthy obese (MUO) individuals [2]. According to the current definition, body mass index (BMI) above 30 kg/m^2^ allows the diagnosis of obesity. Obesity causes worsening of quality of life and poses economic burden for countries worldwide [3]. The prevalence rate of obesity increased by 27.5% between 1980 and 2013 [4].

Cardiovascular diseases (CVDs), such as coronary heart disease and stroke, belong to the most important causes of mortality worldwide [5, 6]. The incidence of CVDs among young people has increased past two decades, and it may be associated with increasing incidence of obesity [7]. Atherosclerosis is the main mechanism leading to CVDs [8]. Oxidative stress is suspected to play an important role in the pathogenesis of atherosclerosis [9].

Oxidative stress results from imbalance between prooxidative and antioxidant factors in favour of prooxidative factors [10]. Representatives of reactive oxygen species (ROS), highly reactive radical or nonradical molecules generated in the course of oxygen metabolism, are superoxide anion, hydroxyl radical, and hydrogen peroxide [11]. Superoxide dismutase (SOD), glutathione peroxidase, and catalase are main antioxidant enzymes in humans [12]. Ascorbic acid, glutathione, beta-carotene, tocopherols, and tocotrienols belong to nonenzymatic antioxidants [13]. Reactive oxygen species can damage macromolecules such as proteins, lipids, and deoxyribonucleic acid (DNA) [14]. Lipid peroxidation assessment by measurement of malondialdehyde blood concentration is a useful marker of oxidative stress [15]. Oxidative stress was shown to be a distinctive feature of obesity [16].

Although there is a distinction between MHO and MUO, research results show that MHO individuals have a greater risk of developing cardiovascular diseases and cardiovascular events than normal weight individuals but less than MUO individuals. Research on the differences in metabolic status between MUO and MHO individuals may contribute to a better understanding of the metabolic heterogeneity phenomenon in obese people and the pathogenesis of cardiovascular disease in the course of obesity. It was recently documented that such abnormalities as increased serum myeloperoxidase (MPO) activity, nitric oxide (NO) formation, and nitrosative damage to proteins are associated with the progression of metabolic disturbances of obesity as well as elevated ONOO—blood concentration may be a valuable predictor of development of hypertension and metabolic syndrome in obese individuals [17].

The purpose of this study was to analyse the association between selected anthropometric and biochemical parameters with oxidative stress in young healthy adults with and without obesity and metabolic disturbances.

## 2. Materials and Methods

Blood samples were taken from healthy volunteers, both descendants of people with premature angiographically confirmed CHD and healthy volunteers from the MAGNETIC study (Metabolic and Genetic Profiling of Young Adults with and without a Family History of Premature Coronary Heart Disease). The detailed recruitment procedure of the MAGNETIC study, which was a case-control study, was published [18]. Individuals were recruited from offspring of patients hospitalized in the Silesian Centre for Heart Diseases due to premature CHD and from untreated people aged 18–36 years without a family history of CHD. From the whole group of recruited volunteers, we selected obese individuals and those with normal weight. The group was divided into two subgroups: metabolically healthy obese (MHO) persons and metabolically unhealthy obese (MUO) persons. The control group constituted of normal weight persons without the metabolic syndrome. Criteria of metabolic health according to Buscemi et al. have been used [19]. Subjects who met at least two of the following conditions are considered metabolically unhealthy: (1) systolic blood pressure (SBP) ≥ 130 mmHg or diastolic blood pressure (DBP) ≥ 85 mmHg or use of antihypertensive medication; (2) triglycerides ≥ 150 mg/dL or use of lipid-lowering medication; (3) high density lipoprotein (HDL) cholesterol < 40 mg/dL (1.0 mmol/L) (men) or < 50 mg/dL (1.2 mmol/L) (women); (4) total cholesterol > 200 mg/dL (5.2 mmol/L) or use of cholesterol-lowering medication; (5) glucose > 100 mg/dL (5.55 mmol/L) or diabetes mellitus type 2. People who met one or no criterion are considered metabolically healthy.

Inclusion criteria for the study group were as follows: (1) age between 18 and 36 years, (2) obesity, (3) no use of medicaments, and (4) agreement for participation in trial. Inclusion criteria for the control group were as follows: (1) age between 18 and 36 years, (2) normal weight, (3) no metabolic syndrome, (4) no use of medicaments, and (5) agreement for participation in the trial. Exclusion criteria for both groups were as follows: (1) age below 18 years or above 36 years, (2) use of medicaments, and (3) acute or chronic disease.

Blood pressure was measured with a certified automatic apparatus. Blood samples were obtained from every individual between 7 am and 9 am, approx. 8–10 h after the last meal. The serum sample was obtained for oxidative stress analyses and frozen immediately at -80 C. Complete blood counts (CBC) were analysed by a Sysmex XE2100 (Sysmex Corporation, Kobe, Japan). Biochemical measurements were analysed by a Cobas 6000 (Roche Diagnostics, Indianapolis, IN, USA) using Roche reagents. The following parameters were assessed: uric acid, total cholesterol, low density lipoprotein (LDL) cholesterol, high density lipoprotein (HDL) cholesterol, triglycerides, and glucose. The measurements were done in a clinical laboratory. In all patients, the body mass, height, BMI, waist-hip ratio (WHR), visceral adipose index (VAI), SBP, and DBP were measured. VAI was calculated from waist circumference (WC), BMI, triglycerides (TG) blood concentration, and high density lipoprotein (HDL) concentration based on the methodology described by Amato et al. [20] according to the following rules:

(1) $$\begin{array}{c} \text{Males}:\text{VAI}=\left(\frac{\text{WC}}{39.68+1.88\times \text{BMI}}\right)\times \left(\frac{\text{TG}}{1.03}\right)\times \left(\frac{1.31}{\text{HDL}}\right),\\ \text{Females}:\text{VAI}=\left(\frac{\text{WC}}{36.58+1.89\times \text{BMI}}\right)\times \left(\frac{\text{TG}}{0.81}\right)\times \left(\frac{1.52}{\text{HDL}}\right). \end{array}$$

The thiol (SH) group serum concentration was measured according to the methodology described by Koster et al. 5,5′-Dithiobis (2-nitrobenzoic acid) (DTNB) is reduced by compounds containing SH groups, yielding the yellow anion derivative, 5-thio-2-nitrobenzoate, absorbing at a wavelength of 412 nm [21]. An automated PerkinElmer analyser was applied. Results have been shown as the thiol group concentration per gram of protein.

Total antioxidant capacity (TAC) was determined by the colorimetric method developed by Erel. Ions 2,2′-azinobis-3-ethylbenzothiazoline-6-sulfonate change colour, and they are measured as the modification in absorbance at 660 nm [22]. This procedure was conducted in an automated PerkinElmer analyser calibrated with Trolox.

The methodology described by Erel has been used to measure serum total oxidative status (TOS). Oxidizing agents in an acidic medium cause the oxidation of ferrous ions to ferric ions. Xylenol orange changes colour in the presence of ferric ions, and it is measured as the change in absorbance at 560 nm [23]. This assay was conducted in an automated analyser (PerkinElmer) calibrated with hydrogen peroxide.

Oxidative stress index (OSI) has been calculated as percentage ratio of TOS to TAC.

Malondialdehyde (MDA) serum concentration was measured fluorometrically according to Ohkawa et al. with modifications. Samples were mixed with 8.1% sodium dodecyl sulfate, 20% acetic acid, and 0.8% 2-thiobarbituric acid. Samples were vortexed and then incubated for one hour at 95 Celsius degrees. In the next step, butanol-pyridine 15 : 1 (*v*/*v*) was added. The mixture was shaken for ten minutes and then centrifuged. The butanol-pyridine layer was measured fluorometrically at 552 nm and 515 nm excitation (PerkinElmer, Waltham, Massachusetts). Tetraethoxypropane was used as the standard. 2-Thiobarbituric acid-reactive substance concentration values are expressed as MDA equivalents [24].

Ceruloplasmin (CER) serum concentration has been determined by the methodology described by Richterich. It is based on the ability of ceruloplasmin to catalyse the oxidation of p-phenylenediamine to the coloured product which can be detected spectrophotometrically [25].

Measurements of lipid hydroperoxides (LPH) concentration were conducted basing on the methodology developed by Sődergren et al. It is associated with colour change of xylenol orange in the presence of ferric ions, followed by the addition of methanol and butylated hydroxytoluene (BHT). The change of absorbance was measured at 560 nm in the Victor X3 automated analyser (PerkinElmer), which was calibrated with hydrogen peroxide [26].

Concentration of lipofuscin (LPS) in erythrocytes has been measured according to Jain as fluorescence of the extraction of erythrocytes in a mixture of isopropanol and chloroform [27]. Results were shown in relative units (RUs).

The activity of superoxide dismutase (SOD) has been measured according to the method published by Oyanagui. In this method, the activity of SOD is negatively proportional to the concentration of coloured product generated in the reaction of nitric ions with naphthalene diamine and sulfanilic acid. Nitric ions are products in the reaction of superoxide ions (generating by xanthine oxidase) with hydroxylamine. Absorbance was measured using an automated PerkinElmer analyser at a wavelength of 550 nm. The enzymatic activity of SOD was expressed in nitric units (NUs). The activity of SOD isoenzymes such as MnSOD and CuZnSOD has been determined using KCN as the inhibitor of the CuZnSOD activity [28].

The experimental protocol has been approved by the Bioethics Committee at the Institute of Occupational Medicine and Environmental Health in Sosnowiec (no. 03/2013) and further extended by the Bioethics Committee at the Medical University of Silesia (no. KNW/0022/KB1/118/18/19).

Statistical analysis has been performed by Statistica 10 (Statsoft, Tibco). The normality of distribution was checked with the Shapiro-Wilk as well as Lilliefors test. Spearman's rank correlation has been performed for the analysis of correlations between determined parameters in the group of all participants and for both men and women separately. Mann–Whitney *U* test has been used to analyse the associations of measured parameters with sex. Kruskal-Wallis ANOVA and post hoc test were used to find differences between MHO, MUO, and metabolically healthy normal weight (MHNW) participants. Statistical tests were performed correcting for multiple comparisons (Dunn's test). Frequencies of categorical values were compared using the *χ*^2^ test. Box plots and respective tests were prepared in the R software.

## 3. Results

422 patients who met inclusion criteria have been enrolled in the study. In this group, 44.8% (189) were men and 55.2% (233) were women. The average age was 27.67 ± 4.48 years in the whole group, 27.69 ± 4.41 years among men and 27.64 ± 4.54 years among women. 16 patients were classified as metabolically healthy obese people (7 women and 9 men). 61 patients met the definition of metabolically unhealthy obese (19 women and 42 men). 345 patients were classified as metabolically healthy with normal weight (MHNW) (207 women and 138 men). Among the study participants, 208 people (49.29%) were offspring of people with angiographically confirmed CHD (98 men and 110 women). 76 participants were current smokers (37 men and 39 women), 47 persons were past smokers (27 men and 20 women), and 298 participants had never smoked (125 men and 173 women). Differences between MHNW, MHO, and MUO individuals in terms of epidemiologic, anthropometric, and biochemical data are presented in Table 1.

There were significant differences between men and women in biochemical parameters and unhealthy behaviour such as alcohol consumption between men and women as well as the parameters of oxidative stress (Table 2). Oxidative stress parameters assessed in the study may be divided into three groups: antioxidant barrier (SH groups, SOD, MnSOD, CuZnSOD, and CER), parameters associated with total antioxidant/oxidant status (TAC, TOS, and OSI), and oxidative damage (MDA, LPH, and LPS). In the whole group of participants, TOS, OSI, and SOD activities (total SOD, MnSOD, and CuZnSOD) and LPS and ceruloplasmin concentrations were significantly higher among women than men. The thiol group concentration per gram of protein was significantly lower among women than men. In both males and females, TAC positively correlated with uric acid concentration.

**Figure 1:**
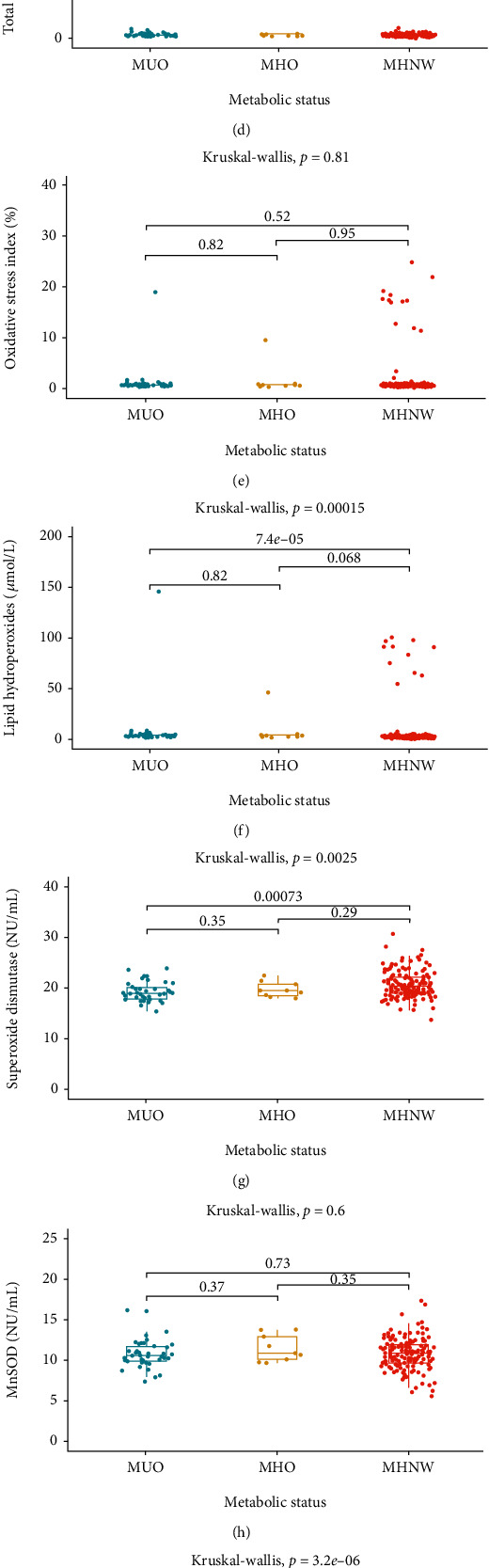
Oxidative stress parameters—differences between men with different metabolic status. MHNW: metabolically healthy normal weight individuals; MHO: metabolically healthy obese individuals; MUO: metabolically unhealthy obese individuals. Data are presented in boxes as median, first, and third quartile values. (a) Thiol group concentration; (b) ceruloplasmin; (c) total antioxidant capacity; (d) total oxidative status; (e) oxidative stress index; (f) lipid hydroperoxides; (g) superoxide dismutase; (h) Mn-dependent superoxide dismutase; (i) Cu- and Zn-dependent superoxide dismutase; (j) lipofuscin; (k) malondialdehyde.

Among men, significant differences associated with metabolic status in LPH and LPS blood concentration and SOD blood activity (total and CuZnSOD, but not MnSOD) were observed. LPH concentration in MUO was higher than in MHNW. LPS concentration was the highest in MHNW and the lowest in MHO. SOD total blood activity and CuZnSOD activity were highest in MHNW (Figure 1, Supplementary table 1). There were no differences between MHO and MUO regarding oxidative stress parameters.

**Figure 2:**
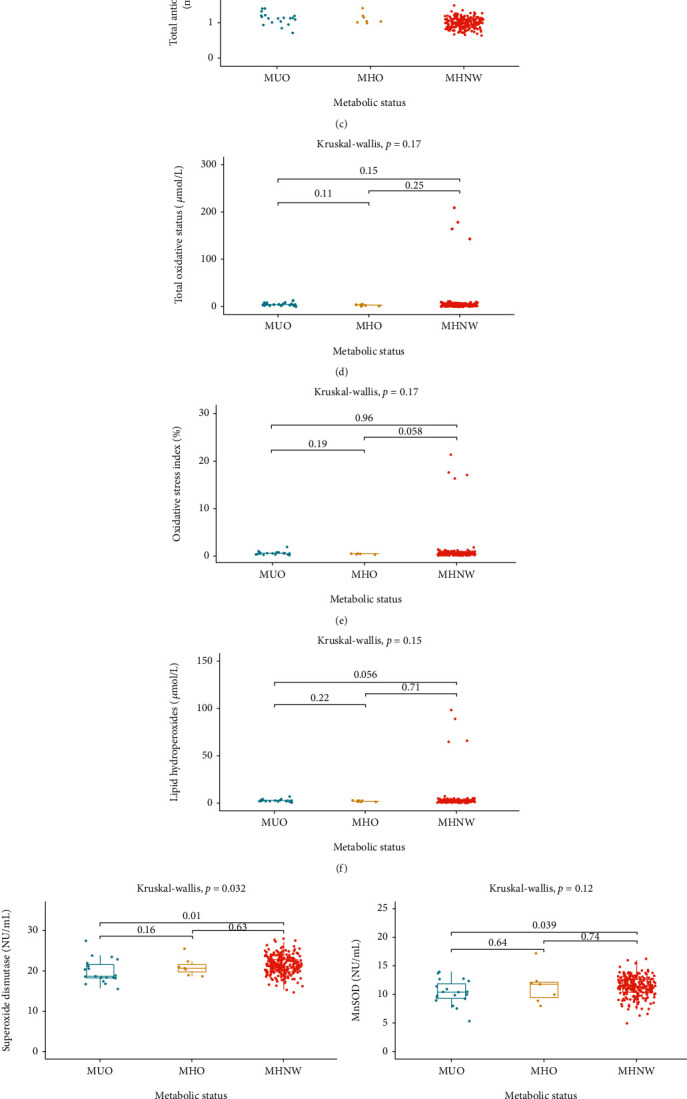
Oxidative stress parameters—differences between women with different metabolic status. MHNW: metabolically healthy normal weight individuals; MHO: metabolically healthy obese individuals; MUO: metabolically unhealthy obese individuals. Data are presented in boxes as median, first, and third quartile values. (a) thiol group concentration; (b) ceruloplasmin; (c) total antioxidant capacity; (d) total oxidative status; (e) oxidative stress index; (f) lipid hydroperoxides; (g) superoxide dismutase; (h) Mn-dependent superoxide dismutase; (i) Cu- and Zn-dependent superoxide dismutase; (j) lipofuscin; (k) malondialdehyde.

Among women, significant differences associated with metabolic status in SH group concentration per gram of protein (PSH), TAC, and total SOD activity (but not MnSOD and CuZnSOD) were observed. TAC was the highest in MUO and the lowest in MHNW. Total SOD activity was higher in MHNW than in MUO (Figure 2, Supplementary table 2).

In men, we observed significant negative correlation between age and TAC, SOD, and CuZnSOD. The LPS negatively correlated with age, total cholesterol, LDL, triglycerides, BMI, WHR, and VAI while positively with HDL. SOD activity negatively correlated with triglycerides, BMI, WHR, and VAI, while opposite correlation was found between the latter parameters and LPH. TAC was negatively correlated with age and HDL cholesterol, while positively with triglycerides, uric acid, and VAI (Table 3).

In women, we observed significant negative correlation between age and SOD and CuZnSOD, while positive with TAC and LPS. The LPH positively correlated with triglycerides, SBP, and VAI. TAC was positively correlated with cholesterol, triglycerides, and uric acid (Table 4).

## 4. Discussion

Our results show that gender differences have a significant impact on parameters related to oxidative stress. In some parameters, we observed a significant trend related to the number of metabolic disturbances from healthy persons with normal weight to metabolically unhealthy obese. Comparing to other studies, our subjects were younger and they were not treated for any disease.

The strength of our study is undoubtedly the participation of young people, without a significant medical history and medication administration. The limitation of our work is the design and the lack of follow-up which could allow us to investigate the relationship between selected parameters of oxidative stress, in the predisposition to develop coronary artery disease. The prospective studies are needed. The study presented by us is a case-control study; therefore, it allows only to determine the presence of specific correlations, not a cause-and-effect relationship. Another limitation of the study is the lack of evaluation of oxidative stress in red blood cells and the lack of oxidative nucleic acid as well as protein damage. Another limitation is the lack of one accepted definition of MHO and MUO; thus, the necessity to choose one of the suggested. We used the criteria according to Buscemi, which take into account parameters such as blood pressure, blood glucose concentration, and plasma lipid profile (TG, TC, and HDL-C). The criteria for the diagnosis of MUO developed by other authors take into account also other parameters. For example, the Karelis criteria and the Wildman criteria include the value of the homeostasis model assessment of insulin resistance (HOMA-IR) index [29], which was not calculated in our study.

### 4.1. Gender Differences in Terms of Oxidative Stress

According to the results of the study conducted on the healthy population by Brunelli et al., the state of oxidative stress is higher in women than in men. In this study, such parameters as diacron reactive oxygen metabolite (dROM) and biological antioxidant potential (BAP) have been assessed [30]. Higher lipid peroxidation among women has been reported in the study conducted by Block et al. [31] In the other study, higher oxidative stress in men than in women measured by blood concentration of the thiobarbituric acid-reacting substances (TBARS) and urinary concentration of 8-iso-PGF2*α* has been documented [32]. Nielsen et al. have shown that MDA concentration is slightly but significantly higher in men than in women [33]. The data available in the literature therefore show discrepancies in the relationship between sex and oxidative stress, which our study also confirms; however, we have also shown higher intensity of oxidative stress in women than in men regarding following parameters: PSH, CER, TOS, SOD, MnSOD, CuZnSOD, and LPS.

Due to the described differences between men and women in terms of the parameters of oxidative stress in our cohort, we decided to analyse the relationship between body weight and metabolic health status and the parameters of oxidative stress separately for women and men.

### 4.2. Oxidative Stress in Association with Body Weight and Metabolic Health

#### 4.2.1. Oxidative Stress Parameters Related to Antioxidant Barrier

Gol et al. studied the differences in the concentration of native thiol groups (-SH) and the total concentration of thiol groups (-SH and -S-S-) between people who lead a sedentary lifestyle, people who are overweight or obese, and people who exercise regularly. There was no significant difference between these groups in the native thiol groups. In terms of the total number of thiol groups, the highest value was found among people who exercise regularly and the lowest among people who lead a sedentary lifestyle [34]. In our study, we assessed the concentration of thiol groups per gram of protein. In women, the value of this parameter was decreased both in MHO and in MUO, which implies the higher intensity of oxidative stress, and was negatively correlated with triglycerides and BMI and there was no association with uric acid, while in men, the parameter positively correlated with triglycerides and uric acid, while negatively with HDL cholesterol concentration. Decreased concentration of reduced thiol groups may be a result of increased oxidative stress in those individuals and indicated different prooxidant and antioxidant mechanisms in men and in women.

Alissa et al. published the results of a study aimed at assessing the relationship between selected parameters of iron metabolism and dietary iron supply and risk factors for coronary heart disease among men from Saudi Arabia. 270 men participated in the study: 130 of whom underwent coronary angiography (<50% stenosis was found) constituted the study group and 140 persons without grounds for suspecting coronary artery disease constituted the control group. There was a significantly higher incidence of such cardiovascular risk factors as diabetes mellitus, hypertension, and metabolic syndrome in the research group. People in the study group were also significantly older than those in the control group (55.6 ± 1.1 vs. 41.2 ± 1.5 years). It was found that in the research group, the concentration of ceruloplasmin in the blood was significantly higher [35]. In our study, no significant differences in the concentration of ceruloplasmin were found between the groups with or without metabolic disturbances; however, we observed that it was significantly higher in women. Perhaps the increase in ceruloplasmin concentration occurs only at a later stage of metabolic disorder progression than in the population we studied.

SOD is one of the most important antioxidant enzymes in the human organism [36]. Baynes and Thorpe suggested that in response to oxidative stress, cells increase the production of this enzyme to prevent mitochondria from oxidative damage [37, 38]. The function of this enzyme is to catalyse the dismutation reaction of the highly reactive superoxide radical anion. SOD occurs in the form of three isoenzymes: cytosolic CuZnSOD, mitochondrial MnSOD, and extracellular SOD (EC-SOD). It has been shown that oxidative stress causes increase in the expression of antioxidant enzymes such as SOD [39]. The data available in the literature on the dependence of SOD activity on body weight and metabolic health status are not unequivocal. Isogawa et al. published data that gave partially similar conclusions to ours. According to the results of their study, SOD activity negatively correlates with BMI. They have shown also that lower SOD activity correlates with increased carotid intima-media thickness which is a well-known indicator of increased cardiovascular risk, but on the other hand, existence of carotid plaque positively correlates with SOD activity [40]. Among the population we studied, the relationship is even stronger in obese individuals with a metabolically unhealthy phenotype and is slightly greater in women than in men. Yubero-Serrano et al. showed that the activity of SOD in patients with two components of metabolic syndrome is significantly lower than in patients with three or more concomitant components of the metabolic syndrome. They have concluded that SOD activity could be the most relevant biomarker of oxidative stress in patients suffering from metabolic syndrome [41]. It is noteworthy that in both above-mentioned studies, mean age of participants was similar and higher than in our study. Isogawa et al. performed their study on the group of patients in which only some of the participants had components of the metabolic syndrome, but Yubero-Serrano et al. enrolled only people with metabolic syndrome. Farah et al. have shown no significant difference in SOD activity between MHO and MUO patients [42]. According to our results, the total blood SOD activity was significantly lower in MUO than in MHNW, both in women (difference 6.37%; *p* = 0.0025) and in men (difference 11.79%; *p* = 0.0321). According to our research, the determination of total blood SOD activity could be a marker of obesity-related metabolic disorders, both in women and in men.

#### 4.2.2. Oxidative Stress Parameters Related to Total Antioxidant/Oxidant Status

The term TAC of plasma or tissue fluid refers to the total capacity to neutralize the prooxidative potential of reactive oxygen species. It is important that the determination of the TAC takes into account the synergistic or antagonistic effect of the various antioxidants present in the plasma or tissue fluid, as opposed to the measurement of the concentration of individual antioxidants [43]. A high fraction of TAC is attributed to uric acid as the endogenous antioxidant molecule [44]. That is why we observed a strong correlation in the whole group.

OSI is the percentage ratio of TOS to TAC. In a study by Nowicki et al., in which people after cardiovascular events (study group) and those without a history of cardiovascular events (control group) participated, no significant difference was found in OSI between the research and the control group, as well as no significant correlation was found between OSI and BMI [45]. Our study also showed no significant difference in terms of OSI between the MUO, MHO, and MHNW groups. Romuk et al. conducted a study to investigate the differences in the parameters of oxidative stress between patients with ischemic and nonischemic cardiomyopathy. It has been found that in patients with ischemic cardiomyopathy, the TAC value is significantly higher and the OSI value is significantly lower. There were no significant differences in TOS. In the study, people with normal body weight or overweight participated [46]. In obese and overweight adolescents, TAC and TOS measured in the plasma and saliva were documented to be higher than in the control group [47]. Higher BMI was shown to be an independent risk factor for the lower total antioxidant status (TAS) which may be correlated with increased carotid intima-media thickness in patients with arterial hypertension [48]. In our study, TAC was significantly higher in obese women with metabolic diseases, and we found an increasing correlation of TAC with increasing metabolic disorders, measured as total cholesterol and triglyceride level. This may suggest that obesity and related metabolic disturbances may contribute to the development of coronary artery disease in a young population. In our results, the TAC among MHO women was 2.91% higher than that among MHNW women, and the TAC value among MUO women was 10.68% higher than that among MHNW women (*p* = 0.0042). Our results suggest that TAC could be a marker of metabolic disorders developing in the course of obesity in women, but more research is needed in this direction.

#### 4.2.3. Oxidative Stress Parameters Related to Oxidative Damage

Simão et al. conducted a study that compared the parameters of oxidative stress in patients who had suffered an ischemic stroke, depending on the coexistence or absence of features of the metabolic syndrome. In the group of patients with metabolic syndrome, a significantly higher concentration of lipid hydroperoxides and a significantly higher value of the oxidative stress index were found. In the entire population studied by us, as well as among men analysed separately, the concentration of lipid hydroperoxides was significantly higher in metabolically obese patients (MUO) but, as already mentioned, without significant differences in OSI [49]. As shown in this study, MUO subjects had increased hydroperoxide lipid levels compared to MHNW and MHO individuals. It has been previously demonstrated that hydroperoxides derivatives are the main primary products of lipid oxidation, particularly in several human inflammatory diseases including diabetes, hypertension, dyslipidaemia, and metabolic syndrome [50, 51].

Lipofuscin is a conglomerate of highly oxidized proteins and lipids considered to be a marker of cell aging. Oxidative stress plays a role in the formation of lipofuscin [52]. There is not a large amount of data elucidating the clinical relevance of LPS measurement in metabolic syndrome. Cazzola et al. have documented that overweight and obesity are associated with increased LPS content in erythrocytes. Taking into account the overall results of this study, it is probably associated with increased lipid peroxidation and the making of aldehydes promoting the formation of cross-links between proteins and phospholipids. Only metabolically healthy females have been enrolled into the study [53]. Our results are therefore not fully comparable as we showed a downward trend for LPS content with increasing metabolic abnormalities. Further studies are needed to better understand the role of LPS as a possible marker of oxidative stress in the course of metabolic disorders.

Research shows that metabolically healthy obesity (MHO) is not a permanent condition. Echouffo-Tcheugui et al. showed that in a four-year follow-up, 43% of MHO women and 46% of MHO men develop disorders that allow them to qualify as metabolically obese patients (MUO) [54]. Moreover, even obese people who are metabolically healthy have an increased health risk. According to the results of the meta-analysis carried out by Zheng et al., MHO individuals have a significantly higher risk of cardiovascular events than metabolically healthy normal weight people (MHNW) (RR 1.5; 95% CI 1.27–1,77) [55]. Bell et al. demonstrated an increased risk of developing type 2 diabetes in this population [56], and Arnlöv et al. showed an increased risk of developing CVD [57]. The state of MHO should therefore not be regarded as completely normal but rather as a transition period between the state of health and the development of overt metabolic syndrome, as well as a condition associated with an increased risk of morbidity.

### 4.3. Can Supplementation of Antioxidants Be a Useful Direction in Therapy?

The results presented in this study show that obese people with metabolic disorders show more disturbances in the parameters of oxidative stress than people with normal body weight without metabolic disorders. The literature suggests the usefulness of many nutraceuticals with antioxidant properties in obese patients, but much of the information on this topic is based mainly on the results of basic research [58]. Recently, the role of isoflavones in the prevention and therapy of type 2 diabetes has been described in detail [59], as well as the properties of phytoestrogens in the context of their use in the prevention of obesity and the resulting disorders [60]. However, it should be noted that this subject is of great interest, and in recent years, the results of clinical trials and meta-analyses evaluating the importance of antioxidant supplementation in the context of the treatment of obesity and related metabolic disorders have been published. Suliburska et al. presented the results of a randomized, double-blind study, which showed that a 3-month supplementation of green tea extract has a positive effect on body weight, lipid profile, blood glucose concentration, and TAS in obese people, as well as on zinc and magnesium metabolism [61]. A systematic review and meta-analysis of clinical trials showed that spirulina supplementation is associated with weight loss in obese subjects [62]. Saffron has documented beneficial effects on waist circumference and fasting blood glucose [63]. The supplementation of certain nutraceuticals with antioxidant properties has a beneficial effect on the metabolic status of obese people and may be considered in the treatment of obese people. However, it should be remembered that such an approach should be of an auxiliary nature and cannot replace a lifestyle change, including a rational diet and exercise, leading to weight loss.

## 5. Summary

Our findings suggest that metabolically unhealthy obese patients have more pronounced oxidative stress parameters comparing to those with normal weight without metabolic disturbances. The significant differences between men and women may imply different mechanisms leading to oxidative stress and its complications. Our results are partially confirmed by the studies that have been conducted so far, which, however, mainly concerned patients with already developed disease entities, which are complications of obesity.

An important achievement of our research is the demonstration that the total SOD activity is statistically significantly lower in MUO compared to MHNW in both women and men, while the TAC value increases significantly in MUO women. Our study involved young people, untreated for chronic diseases; therefore, these parameters of oxidative stress could be considered for use in clinical practice as early markers of the development of metabolic disorders with weight gain. More research is needed. These results show that obesity is always a pathological condition, despite the lack of specific disease entities already developed.

## Figures and Tables

**Table 1:** Differences between MHNW, MHO, and MUO in terms of epidemiologic, anthropometric, and biochemical data.

Variable	MHNW		MHO		MUO		MHNW vs. MHO^∗^	MHNW vs. MUO^∗^	MHO vs. MUO^∗^
	Mean (SD) (*n* (%))	*N*	Mean (SD) (*n* (%))	*N*	Mean (SD) (*n* (%))	*N*			
Age (years)	27.05 (4.44)	344	30.53 (2.91)	16	30.22 (3.73)	61	0.0052	<0.001	ns
Females (%)	206 (59.9%)	344	7 (43.8%)	16	19 (31.2%)	61	ns^#^	ns^#^	ns^#^
Alcohol (yes)	260 (75.4%)	345	13 (81.2%)	16	42 (68.8%)	61	ns^#^	ns^#^	ns^#^
Smoking (i) Current vs. (ii) Past (iii) Never	56 (16.33%) 257 (74.93%) 30 (8.75%)	343	4 (25%) 8 (50%) 4 (25%)	16	16 (26.23%) 32 (52.46%) 13 (21.31%)	61	ns^#^	ns^#^	ns^#^
Family history of premature coronary heart disease (CHD) (yes)	154 (44.6%)	345	10 (62.5%)	16	44 (72.1%)	61	ns^#^	ns^#^	ns^#^
Family history of type 2 diabetes (T2D) (yes)	177 (51.3%)	345	7 (43.7%)	16	38 (62.3%)	61	ns^#^	ns^#^	ns^#^
Total cholesterol (TC) (mmol/L)	4.68 (0.87)	344	4.57 (0.69)	16	5.67 (1.07)	61	ns	<0.001	0.0015
High density lipoprotein cholesterol (HDL-C) (mmol/L)	1.75 (0.41)	344	1.44 (0.35)	16	1.12 (0.25)	61	0.0065	<0.001	0.033
Low density lipoprotein cholesterol (LDL-C) (mmol/L)	2.66 (0.78)	344	2.79 (0.65)	16	3.76 (0.94)	61	ns	<0.001	0.0030
Triglycerides (TG) (mmol/L)	0.83 (0.36)	344	1.1 (0.36)	16	2.56 (2.27)	61	0.014	<0.001	0.019
Glucose (mmol/L)	4.87 (0.37)	344	5.03 (0.3)	16	5.43 (0.59)	61	ns	<0.001	ns
Uric acid (UA) (*μ*mol/L)	277.65 (63.71)	344	332.13 (73.84)	16	381.15 (61.74)	61	0.014	<0.001	ns
Body mass index (BMI) (kg/m^2^)	21.74 (1.79)	344	32.27 (1.83)	16	33.77 (2.69)	61	ns	<0.001	ns
Waist-hip ratio (WHR)	1.1 (5.45)	338	0.89 (0.05)	16	0.94 (0.08)	60	<0.001	<0.001	ns
VAI	1.06 (5.47)	338	1.27 (0.56)	16	3.88 (4.75)	60	<0.001	<0.001	0.040
Systolic blood pressure (SBP) (mmHg)	122.42 (12.0)	334	133.47 (22.93)	15	138.05 (14.86)	60	ns	<0.001	ns
Diastolic blood pressure (DBP) (mmHg)	76.63 (9.46)	335	78.67 (11.36)	15	85.81 (10.01)	58	ns	<0.001	0.023

Mean (SD): mean value (standard deviation) calculated for quantitative features; *n* (%): the number of people (the percentage of this number in relation to the subgroup) given in the case of qualitative features; *N*: size of the subgroup; MHNW: metabolically healthy normal weight individuals; MHO: metabolically healthy obese individuals; MUO: metabolically unhealthy obese individuals; ns: not significant. ^∗^*p* values according to Kruskal-Wallis test; ^#^*p* values according to *χ*^2^ test.

**Table 2:** Differences between men and women in terms of biochemical and oxidative stress parameters as well as epidemiologic and anthropometric data.

Variable	Men Mean (SD) (*n* (%))/median (Q1; Q3)	*N*	Women Mean (SD) (*n* (%))/median (Q1; Q3)	*N*	*p* value *U* Mann–Whitney/*χ*^2∗^
Age (years)	27.69 (4.41)	189	27.6374 (4.54)	233	ns
Alcohol (yes)	159 (84%)	189	155 (67%)	232	0.001^∗^
Smoking (i) Current vs. (ii) Past (iii) Never	37 (20%) 27 (14%) 125 (66%)	189	39 (17%) 20 (9%) 172 (74%)	231	ns^∗^
Family history of premature coronary heart disease (CHD) (yes)	98 (52%)	189	109 (47%)	232	ns^∗^
Family history of type 2 diabetes (T2D) (yes)	91 (48%)	189	130 (56%)	232	ns^∗^
Total cholesterol (TC) (mmol/L)	4.83 (1.06)	189	4.82 (0.86)	233	ns^∗^
Low density lipoprotein cholesterol (LDL-C) (mmol/L)	2.98 (0.96)	189	2.70 (0.81)	233	0.0072
High density lipoprotein cholesterol (HDL-C) (mmol/L)	1.44 (0.35)	189	1.82 (0.44)	232	<0.001
Triglycerides (TG) (mmol/L)	1.32 (1.52)	189	0.91 (0.50)	232	0.00049
Glucose (mmol/L)	5.06 (0.46)	188	4.87 (0.43)	232	<0.001
Uric acid (UA) (*μ*mol/L)	342.63 (64.03)	189	255.67 (55.81)	233	<0.001
Body mass index (BMI) (kg/m^2^)	25.52 (5.20)	189	22.54 (4.27)	233	<0.001
Systolic blood pressure (SBP) (mmHg)	130.89 (14.64)	189	120.35 (11.79)	233	<0.001
Diastolic blood pressure (DBP) (mmHg)	79.76 (10.58)	189	76.55 (9.45)	233	0.0022
Thiol group concentration (PSH) (*μ*mol/g protein)	4.39 (4.00; 4.70)	189	4.00 (3.60; 4.20)	233	<0.001
Ceruloplasmin (CER) (mg/dL)	36.10 (31.80; 41.40)	189	45.10 (38.70; 55.10)	233	<0.001
Total antioxidant capacity (TAC) (mmol/L)	1.02 (0.950; 1.130)	189	1.04 (0.940; 1.130)	233	0.905
Total oxidative status (TOS) (*μ*mol/L)	4.70 (3.600; 6.100)	189	5.300 (4.40; 6.950)	232	0.0024
Oxidative stress index (OSI) (%)	0.45 (0.36; 0.61)	189	0.51 (0.41; 0.67)	232	0.0034
Lipid hydroperoxides (LPH) (*μ*mol/L)	2.39 (1.90; 3.10)	188	2.50 (2.00; 3.10)	232	0.36
Superoxide dismutase (SOD) (NU/mL)	19.63 (18.44; 21.651)	189	21.10 (19.70; 22.70)	233	<0.001
MnSOD (NU/mL)	10.80 (9.77; 11.90)	189	11.40 (10.10; 12.50)	233	0.0015
CuZnSOD (NU/mL)	9.066 (8.10; 10.30)	189	9.80 (8.60; 11.00)	233	0.00026
Lipofuscin (LPS) (RU/L)	190.30 (114.01; 302.30)	189	275.20 (196.40; 350.20)	233	<0.001
Malondialdehyde (MDA) (*μ*mol/L)	1.66 (1.29; 2.06)	186	1.690 (1.310; 2.110)	233	0.52

Mean (SD): mean value (standard deviation) calculated for quantitative features; *n* (%): the number of people (the percentage of this number in relation to the subgroup) given in the case of qualitative features; *N*: size of the subgroup; MHNW: metabolically healthy normal weight individuals; MHO: metabolically healthy obese individuals; MUO: metabolically unhealthy obese individuals; ns: not significant; Q1: first quartile; Q3: third quartile. ^∗^*χ*^2^ test.

**Table 3:** Spearman correlations *R* between oxidative stress parameters in men and anthropometric and biochemical measurements.

Variables	Thiol group concentration (PSH)	Ceruloplasmin (CER)	Total antioxidant capacity (TAC)	Total oxidative status (TOS)	Oxidative stress index (OSI)	Lipid hydroperoxides (LPH)	Superoxide dismutase (SOD)	MnSOD	CuZnSOD	Lipofuscin (LPS)	Malondialdehyde (MDA)
Age	-0.026	-0.034	-0.198^∗∗^	-0.112	-0.054	-0.022	-0.156^∗^	-0.013	-0.260^∗∗∗^	-0.217^∗∗^	0.023
Total cholesterol (TC)	0.0039	-0.00054	-0.028	0.0064	0.022	0.108	-0.136	-0.0090	-0.209^∗∗^	-0.256^∗∗∗^	-0.012
Low density lipoprotein cholesterol (LDL-C)	0.0135	0.046	0.0030	-0.022	-0.012	0.0782	-0.12	-0.0202	-0.17	-0.246^∗∗∗^	-0.0034
High density lipoprotein cholesterol (LDL-C)	-0.187^∗^	-0.128	-0.186^∗^	-0.111	-0.0625	-0.271^∗∗∗^	0.116	0.0276	0.171^∗^	0.182^∗^	-0.0061
Triglycerides	0.191^∗∗^	0.014	0.173^∗^	0.12	0.070	0.224^∗∗^	-0.184^∗^	-0.0832	-0.216^∗∗^	-0.256^∗∗∗^	-0.029
Glucose	0.089	-0.075	0.018	-0.048	-0.024	0.011	-0.037	0.044	-0.101	-0.116	0.030
Uric acid (UA)	0.144^∗^	0.148^∗^	0.362^∗∗∗∗^	-0.027	-0.12	0.102	-0.0065	0.053	-0.093	-0.198^∗∗^	-0.014
Body mass index (BMI)	0.063	-0.041	-0.017	0.040	0.044	0.272^∗∗∗^	-0.237^∗∗^	-0.05	-0.299^∗∗∗^	-0.313^∗∗∗^	0.045
Systolic blood pressure (SBP)	0.087	0.024	-0.108	0.0784	0.11	0.218^∗∗^	-0.0296	0.143	-0.187^∗^	-0.0434	-0.0273
Diastolic blood pressure (DBP)	0.088	0.096	-0.142	0.0656	0.0742	0.176^∗^	0.0282	0.204^∗∗^	-0.201^∗∗^	-0.122	-0.036
Waist-hip ratio (WHR)	0.049	0.054	0.105	-0.056	-0.086	0.101	-0.241^∗∗∗^	-0.039	-0.329^∗∗∗^	-0.160^∗^	0.089
Visceral adipose index (VAI)	0.188^∗^	0.068	0.207^∗∗^	0.112	0.052	0.239^∗∗^	-0.202^∗∗^	-0.083	-0.253^∗∗∗^	-0.237^∗∗^	0.0034

^∗^ *p* < 0.05; ^∗∗^*p* < 0.01; ^∗∗∗^*p* < 0.001.

**Table 4:** Spearman correlations *R* between oxidative stress parameters and anthropometric and biochemical measurements in women.

Variables	Thiol group concentration (PSH)	Ceruloplasmin (CER)	Total antioxidant capacity (TAC)	Total oxidative status (TOS)	Oxidative stress index (OSI)	Lipid hydroperoxides (LPH)	Superoxide dismutase (SOD)	MnSOD	CuZnSOD	Lipofuscin (LPS)	Malondialdehyde (MDA)
Age	-0.102	0.138^∗^	0.131^∗^	0.0095	-0.037	0.0217	-0.155^∗^	-0.038	-0.177^∗∗^	0.131^∗^	0.091
Total cholesterol (TC)	-0.070	0.234^∗∗∗^	0.134^∗^	0.143^∗^	0.075	0.109	-0.071	-0.092	-0.013	0.010	0.157^∗^
Low density lipoprotein cholesterol (LDL-C)	-0.032	0.105	0.114	0.027	-0.022	0.021	-0.159^∗^	-0.189^∗∗^	-0.048	-0.036	0.121
High density lipoprotein cholesterol (HDL-C)	-0.050	0.103	-0.051	0.077	0.104	0.038	0.069	0.086	0.030	0.111	0.043
Triglycerides	-0.160^∗^	0.386^∗∗∗^	0.151^∗^	0.246^∗∗∗^	0.131^∗^	0.261^∗∗∗^	-0.104	-0.128	-0.035	0.028	0.212^∗∗^
Glucose	-0.040	-0.113	0.067	0.021	-0.0011	0.038	-0.0091	0.019	-0.0504	0.099	0.0205
Uric acid (UA)	-0.058	-0.053	0.447^∗∗∗^	-0.029	-0.214^∗∗^	-0.018	0.040	0.016	0.022	0.041	0.0050
Body mass index (BMI)	-0.146^∗^	0.142^∗^	0.084	0.029	-0.021	0.090	-0.072	-0.076	-0.061	-0.030	0.047
Systolic blood pressure (SBP)	-0.062	0.232^∗∗∗^	-0.010	0.134^∗^	0.12	0.17^∗∗^	0.026	0.101	-0.077	-0.025	-0.012
Diastolic blood pressure (DBP)	-0.061	0.205^∗∗^	-0.057	0.101	0.11	0.12	0.0077	0.054	-0.079	0.069	0.054
Waist-hip ratio (WHR)	0.0089	-0.013	0.120	-0.027	-0.076	-0.018	-0.024	-0.0099	-0.053	-0.045	0.014
Visceral adipose index (VAI)	-0.110	0.287^∗∗∗^	0.126	0.154^∗^	0.057	0.181^∗∗^	-0.1099	-0.133^∗^	-0.041	-0.019	0.147^∗^

^∗^ *p* < 0.05; ^∗∗^*p* < 0.01; ^∗∗∗^*p* < 0.001.

## Data Availability

The data presented in this study are available on request from the corresponding author.

## Abbreviations

BMI: Body mass index
CBC: Complete blood count
CER: Ceruloplasmin
CHD: Coronary heart disease
CI: Confidence interval
CuZnSOD: Copper- and zinc-containing superoxide dismutase
CVD: Cardiovascular disease
DBP: Diastolic blood pressure
EC-SOD: Extracellular superoxide dismutase
HDL: High density lipoprotein
HDL-C: High density lipoprotein cholesterol
HOMA-IR: Homeostasis model assessment of insulin resistance
HR: Hazard ratio
LDL: Low density lipoprotein
LDL-C: Low density lipoprotein cholesterol
LPH: Lipid hydroperoxides
LPS: Lipofuscin
MDA: Malondialdehyde
MHNW: Metabolically healthy normal weight
MHO: Metabolically healthy obese/obesity
MnSOD: Manganese-containing superoxide dismutase
MPO: Myeloperoxidase
MUO: Metabolically unhealthy obese/obesity
NU: Nitric unit
OSI: Oxidative stress index
oxLDL: Oxidized low density lipoproteins
PSH: Thiol group concentration per gram protein
ROS: Reactive oxygen species
RR: Relative risk
RU: Relative unit
SBP: Systolic blood pressure
SOD: Superoxide dismutase
TAC: Total antioxidant capacity
TAS: Total antioxidant status
TBARS: Thiobarbituric acid-reacting substances
TC: Total cholesterol
TG: Triglycerides
TOS: Total oxidative status
UA: Uric acid
VAI: Visceral adipose index
WC: Waist circumference
WHR: Waist-hip ratio.

## Supplementary Materials

Supplementary Materials Supplementary Table 1: oxidative stress parameters—differences in men with different metabolic status. Supplementary Table 2: oxidative stress parameters—differences in women with different metabolic status.
