# Supplementary material for: Oxidative Stress in Association with Metabolic Health and Obesity in Young Adults
Source: Oxid Med Cell Longev. 2021 Jun 26;2021:9987352. doi: 10.1155/2021/9987352 (PMC8257366; doi:10.1155/2021/9987352)
Supplement: Supplementary Materials — Supplementary Table 1: oxidative stress parameters—differences in men with different metabolic status. Supplementary Table 2: oxidative stress parameters—differences in women with different metabolic status. [file 9987352.f1.zip › Jakubiak et al. Supplementary Table 1..docx]

**Supplementary Table 1.** Oxidative stress parameters – differences in men with different metabolic status.

| Variable | Males All  Median (Q1; Q3) | N | MHNW Males  Median (Q1; Q3) | N | MHO Males  Median (Q1; Q3) | % difference vs. MHNW | N | MUO Males  Median (Q1; Q3) | % difference vs. MHNW | N | p value  Kruskal-Wallis | MHNW vs. MHO | MHNW vs. MUO | MHO vs. MUO |
| --- | --- | --- | --- | --- | --- | --- | --- | --- | --- | --- | --- | --- | --- | --- |
| Thiol group concentration (PSH) [μmol/g protein] | 4.39 (4.00; 4.70) | 189 | 4.30 (4.00; 4.60) | 138 | 4.40 (4.28; 4.66) | 2.35% | 9 | 4.50 (4.15; 4.87) | 4.72% | 42 | 0.12 |  |  |  |
| Ceruloplasmin (CER) [mg/dL] | 36.10 (31.80; 41.40) | 189 | 35.95 (31.80; 40.80) | 138 | 32.95 (29.36; 38.37) | -8.33% | 9 | 37.80 (32.40; 43.20) | 5.16% | 42 | 0.45 |  |  |  |
| Total antioxidant capacity (TAC) [mmol/L] | 1.02 (0.95; 1.13) | 189 | 1.016 (0.95; 1.16) | 138 | 0.976 (0.96; 1.011) | -3.94% | 9 | 1.021 (0.95; 1.088) | 0.49% | 42 | 0.596 |  |  |  |
| Total oxidative status (TOS) [μmol/L] | 4.70 (3.60; 6.10) | 189 | 4.60 (3.60; 6.00) | 138 | 4.42 (3.52; 6.40) | -3.87% | 9 | 4.92 (3.47; 6.45) | 6.98% | 42 | 0.88 |  |  |  |
| Oxidative stress index (OSI) [%] | 0.45 (0.36; 0.61) | 189 | 0.44 (0.36; 0.59) | 138 | 0.43 (0.36; 0.64) | -3.56% | 9 | 0.50 (0.36; 0.63) | 14.12% | 42 | 0.81 |  |  |  |
| Lipid hydroperoxides (LPH) [μmol/L] | 2.39 (1.90; 3.10) | 188 | 2.20 (1.70; 2.90) | 137 | 3.00 (2.30; 3.60) | 36.36% | 9 | 2.85 (2.60; 3.80) | **29.79%** | 42 | 0.00015 |  | 0.000074 |  |
| Superoxide dismutase (SOD) [NU/mL] | 19.63 (18.44; 21.65) | 189 | 20.10 (18.80; 22.10) | 138 | 19.26 (18.45; 20.60) | -4.17% | 9 | 18.82 (17.73; 20.05) | **-6.37%** | 42 | 0.0025 |  | 0.00073 |  |
| MnSOD [NU/mL] | 10.80 (9.77; 11.90) | 189 | 10.87 (9.61; 11.90) | 138 | 10.85 (10.11; 12.90) | -0.22% | 9 | 10.55 (9.94; 11.70) | -2.99% | 42 | 0.60 |  |  |  |
| CuZnSOD [NU/mL] | 9.07 (8.10; 10.30) | 189 | 9.50 (8.50; 10.80) | 138 | 8.42 (7.062; 9.15) | **-11.35%** | 9 | 8.15 (7.30; 9.05) | **-14.21%** | 42 | < 0.001 | 0.0095 | 0.0000046 |  |
| Lipofuscin (LPS) [RU/L] | 190.30 (114.01; 302.30) | 189 | 229.10 (138.51; 320.50) | 138 | 94.16 (88.41; 244.88) | **-58.9%** | 9 | 120.24 (86.79; 171.15) | **-47.51%** | 42 | < 0.001 | 0.021 | 0.000034 |  |
| Malondialdehyde (MDA) [μmol/L] | 1.66 (1.29; 2.06) | 186 | 1.666 (1.30; 2.03) | 136 | 1.44 (0.86; 2.13) | -13.38% | 9 | 1.61 (1.23; 2.06) | -3.60% | 41 | 0.77 |  |  |  |

N – size of the subgroup; MHNW – metabolically healthy normal weight individuals; MHO – metabolically healthy obese individuals; MUO – metabolically unhealthy obese individuals; ns – not significant; Q1 – first quartile; Q3 – third quartile
