# Supplementary material for: Oxidative Stress in Association with Metabolic Health and Obesity in Young Adults
Source: Oxid Med Cell Longev. 2021 Jun 26;2021:9987352. doi: 10.1155/2021/9987352 (PMC8257366; doi:10.1155/2021/9987352)
Supplement: Supplementary Materials — Supplementary Table 1: oxidative stress parameters—differences in men with different metabolic status. Supplementary Table 2: oxidative stress parameters—differences in women with different metabolic status. [file 9987352.f1.zip › Jakubiak et al. Supplementary Table 2..docx]

**Supplementary Table 2.** Oxidative stress parameters – differences in women with different metabolic status.

| Variable | All  Females  Median (Q1; Q3) | N | MHNW Females  Median (Q1; Q3) | N | MHO Females  Median (Q1; Q3) | % difference vs. MHNW | N | MUO Females  Median (Q1; Q3) | % difference vs. MHNW | N | p value  Kruskal-Wallis | MHNW vs. MHO | MHNW vs. MUO | MHO vs. MUO |
| --- | --- | --- | --- | --- | --- | --- | --- | --- | --- | --- | --- | --- | --- | --- |
| Thiol group concentration (PSH) [μmol/g protein] | 4.00 (3.60; 4.20) | 233 | 4.00 (3.60; 4.30) | 207 | 3.60 (3.30; 3.70) | -10.00 | 7 | 3.80 (3.50; 4.20) | -5.00% | 19 | 0.048 | 0.021 |  |  |
| Ceruloplasmin (CER) [mg/dL] | 45.10 (38.70; 55.10) | 233 | 44.60 (38.50; 57.70) | 207 | 48.80 (44.40; 53.00) | 9.42% | 7 | 45.90 (41.00; 54.30) | 2.91% | 19 | 0.52 |  |  |  |
| Total antioxidant capacity (TAC) [mmol/L] | 1.04 (0.94; 1.13) | 233 | 1.03 (0.92; 1.12) | 207 | 1.06 (1.03; 1.21) | 2.91% | 7 | 1.14 (1.03; 1.21) | **10.68%** | 19 | 0.0042 |  | 0.0038 |  |
| Total oxidative status (TOS) [μmol/L] | 5.30 (4.40; 6.95) | 232 | 5.25 (4.40; 7.00) | 206 | 4.40 (3.70; 6.00) | -16.19% | 7 | 5.90 (4.90; 7.10) | 12.38% | 19 | 0.17 |  |  |  |
| Oxidative stress index (OSI) [%] | 0.51 (0.41; 0.67) | 232 | 0.51 (0.42; 0.70) | 206 | 0.42 (0.31; 0.51) | -17.89% | 7 | 0.52 (0.395; 0.68) | 2.64% | 19 | 0.17 |  |  |  |
| Lipid hydroperoxides (LPH) [μmol/L] | 2.50 (2.00; 3.10) | 232 | 2.40 (2.00; 3.10) | 206 | 2.50 (1.70; 2.70) | 4.17% | 7 | 2.60 (2.50; 3.30) | 8.33% | 19 | 0.15 |  |  |  |
| Superoxide dismutase (SOD) [NU/mL] | 21.10 (19.70; 22.70) | 233 | 21.20 (19.80; 22.90) | 207 | 20.80 (19.10; 22.40) | -1.89% | 7 | 18.70 (18.30; 22.00) | **-11.79%** | 19 | 0.032 |  | 0.028 |  |
| MnSOD [NU/mL] | 11.40 (10.10; 12.50) | 233 | 11.40 (10.30; 12.70) | 207 | 11.80 (9.00; 12.30) | 3.51% | 7 | 10.40 (9.30; 12.40) | -8.77% | 19 | 0.12 |  |  |  |
| CuZnSOD [NU/mL] | 9.80 (8.60; 11.00) | 233 | 9.90 (8.80; 11.20) | 207 | 9.00 (8.50; 11.10) | -9.09% | 7 | 9.00 (7.80; 10.40) | -9.09% | 19 | 0.095 |  |  |  |
| Lipofuscin (LPS) [RU/L] | 275.20 (196.40; 350.20) | 233 | 284.50 (196.40; 355.40) | 207 | 269.10 (193.30; 382.30) | -5.41% | 7 | 206.60 (175.70 (278.40) | -27.38% | 19 | 0.106 |  |  |  |
| Malondialdehyde (MDA) [μmol/L] | 1.69 (1.31; 2.11) | 233 | 1.72 (1.30; 2.11) | 207 | 1.48 (1.27; 1.77) | -13.95% | 7 | 1.64 (1.36; 2.45) | -4.65% | 19 | 0.50 |  |  |  |

N – size of the subgroup; MHNW – metabolically healthy normal weight individuals; MHO – metabolically healthy obese individuals; MUO – metabolically unhealthy obese individuals; ns – not significant; Q1 – first quartile; Q3 – third quartile
